# Risk of severe COVID-19 infection among adults with prior exposure to children

**DOI:** 10.1073/pnas.2204141119

**Published:** 2022-07-27

**Authors:** Matthew D. Solomon, Gabriel J. Escobar, Yun Lu, David Schlessinger, Jonathan B. Steinman, Lawrence Steinman, Catherine Lee, Vincent X. Liu

**Affiliations:** ^a^Division of Research, Kaiser Permanente Northern California, Oakland, CA 94612;; ^b^Department of Cardiology, Kaiser Oakland Medical Center, Oakland, CA 94611;; ^c^Department of Pediatrics, Columbia University, New York, NY 10032;; ^d^Department of Pediatrics, Stanford University, Stanford, CA 94305;; ^e^Department of Neurology, Stanford University, Stanford, CA 94305;; ^f^Department of Neurological Sciences, Stanford University, Stanford, CA 94305

**Keywords:** coronavirus, cross-immunity, COVID-19, infectious diseases, public health

## Abstract

Susceptibility and severity of COVID-19 infection vary widely. Prior exposure to endemic coronaviruses, common in young children, may protect against SARS-CoV-2. We evaluated risk of severe COVID-19 among adults with and without exposure to young children in a large, integrated healthcare system. Adults with children 0–5 years were matched 1:1 to adults with children 6–11 years, 12–18 years, and those without children based upon a COVID-19 propensity score and risk factors for severe COVID-19. COVID-19 infections, hospitalizations, and need for intensive care unit (ICU) were assessed in 3,126,427 adults, of whom 24% (N = 743,814) had children 18 years or younger, and 8.8% (N = 274,316) had a youngest child 0–5 years. After 1:1 matching, propensity for COVID-19 infection and risk factors for severe COVID-19 were well balanced between groups. Rates of COVID-19 infection were slightly higher for adults with exposure to older children (incident risk ratio, 1.09, 95% confidence interval, [1.05–1.12] and IRR 1.09 [1.05–1.13] for adults with children 6–11 and 12–18, respectively), compared to those with children 0–5 years, although no difference in rates of COVID-19 illness requiring hospitalization or ICU admission was observed. However, adults without exposure to children had lower rates of COVID-19 infection (IRR 0.85, [0.83–0.87]) but significantly higher rates of COVID-19 hospitalization (IRR 1.49, [1.29–1.73]) and hospitalization requiring ICU admission (IRR 1.76, [1.19–2.58]) compared to those with children aged 0–5. In a large, real-world population, exposure to young children was associated with less severe COVID-19 illness. Endemic coronavirus cross-immunity may play a role in protection against severe COVID-19.

## Significance

Epidemiologic data consistently show strong protection for young children against severe COVID-19 illness. Several mechanisms have been proposed to explain this phenomenon, including cross-reactive immunity—in which prior exposure to non-SARS-CoV-2 coronaviruses that commonly infect children confers some resistance to severe COVID-19 illness. We identified 3,126,427 adults (24% [N = 743,814] with children ≤18, and 8.8% [N = 274,316] with youngest child 0–5 years) to assess whether parents of young children—who have high exposure to non-SARS-CoV-2 coronaviruses—may also benefit from potential cross-immunity. In a large, real-world population, exposure to young children was strongly associated with less severe COVID-19 illness, after balancing known COVID-19 risk factors. This epidemiologic signal suggests endemic coronavirus cross-immunity may play a role in protection against severe COVID-19 outcomes.

Substantial interindividual variability in susceptibility to severe illness following infection with severe acute respiratory syndrome-coronavirus 2 (SARS-CoV-2) has been one of the salient features of the COVID-19 pandemic. While cardiometabolic comorbidities, body mass index (BMI), socioeconomic status, and certain types of immunosuppression are well-established risk factors for susceptibility to severe illness following SARS-CoV-2 infection, age is one of the strongest known modifiers of COVID-19 risk, with increasing case fatality across decades of life (1, 2). Epidemiologic data consistently show strong protection for young children against hospitalization and severe illness due to COVID-19 (3). Several mutually compatible explanations have been proposed to explain this phenomenon, including cross-reactive immunity—in which prior exposure to non-SARS-CoV-2 coronaviruses that commonly infect children confers some resistance to infection and severe illness from COVID-19 (4).

How this potential cross-immunity may translate to adults’ response to COVID-19 infection remains poorly understood. Studies on samples banked prior to the COVID-19 pandemic or on SARS-CoV-2-uninfected donors documented both cell-mediated (4–8) and humoral immunity (4, 9, 10) to SARS-CoV-2 in subjects with prior “common-cold coronavirus” exposure, as well as more distant SARS infection (11). The presence of antibodies recognizing SARS-CoV-2 was highest in children and adolescents, in line with the known peak of exposure to common cold coronaviruses across age groups (9, 12, 13). Although it remains unclear how effective cross-reactive antibodies are at neutralizing SARS-CoV-2 (14), recent data suggest potentially potent protection from preexisting antibodies and preexisting memory T-cell responses (4). Other studies have demonstrated that recent viral upper respiratory infection may limit SARS-CoV-2 replication through the induction of innate immune machinery (15). However, it has so far been challenging to determine whether recent or distant past exposure to viruses—including non-COVID-19 coronaviruses—that cause mild upper respiratory infections truly confers protection from severe COVID-19 illness.

Given the SARS-CoV-2 predilection to cause more severe illness in adults, we sought to examine whether evidence of cross-immunity could be detected in a large population sample. Taking advantage of information systems permitting linkage of records of adult and child members within a large, integrated health care delivery system, we explored the association between possible previous exposure to endemic coronaviruses and subsequent development of severe COVID-19 by examining outcomes among adults with exposure to children of different age groups, since adults with children less than 5 y have increased exposure to prior coronaviruses and other upper respiratory viruses through their proximity to young children (12, 13, 16–18).

## Methods

### Study Population.

Our base population consisted of members in Kaiser Permanente Northern California (KPNC), a large, integrated healthcare delivery system providing comprehensive inpatient, emergency department, and outpatient care for >4.5 million members in 21 hospitals and >255 outpatient clinics across Northern California. The KPNC membership is highly representative of the surrounding local and statewide population in terms of age, gender and race/ethnicity (19).

This study was approved by the KPNC institutional review board. Waiver of informed consent was obtained due to the nature of the study.

### Study Sample.

We identified all persons ≥18 y old who were active health plan members as of February 1, 2020, with no continuous gaps in coverage between February 1, 2019 through January 31, 2021 within KPNC. For each adult member, we identified all children less than 18 y old subscribed on the same health insurance plan. We excluded adults who were not active KPNC health plan members during the study period or who had unknown gender.

### Exposure.

We categorized adults into mutually exclusive groups based upon the ages of the children in their subscriber household as of February 1, 2020 (roughly the beginning of the COVID-19 pandemic): 1) adults with children ages 0–5 in the household (Study Population), 2) adults with children ages 6–11 in the household (Comparator Group 1), 3) adults with children ages 12–18 in the household (Comparator Group 2), and 4) adults with no children in the household (Comparator Group 3). We placed members in the group corresponding to their youngest child. For example, if a member had a 1-year-old child and an 8-year-old child on February 1, 2020, they were classified in the group of adults with children aged 0–5 in the household. Follow-up for all patients continued from the index date of February 1, 2020 through January 31, 2021.

### Covariates.

Demographic information was obtained through health plan enrollment files. In addition, based on previously validated algorithms (20, 21), we used health plan administrative and clinical data, pharmacy databases, and laboratory databases to identify the following comorbidities before the index date of February 20, 2020: hypertension; diabetes mellitus; chronic pulmonary disease; peripheral vascular disease; chronic kidney disease; malignancy; cerebrovascular disease; congestive heart failure; rheumatologic disease; myocardial infarction; dementia; metastatic solid tumor; liver disease; hemiplegia or paraplegia; HIV/AIDS; and peptic ulcer disease.

### Outcomes.

The primary outcomes were 1) SARS-CoV-2 infection based upon PCR testing, 2) hospitalization for COVID-19, and 3) hospitalization for COVID-19 requiring treatment in the intensive care unit (ICU). KPNC maintains a validated database for all three COVID-19 outcomes (20, 22–24).

### Propensity-Matching.

To improve comparability between the Study and Comparator groups and balance baseline characteristics for factors related to the risk of severe COVID-19 illness, we developed 1:1 propensity-matched cohorts between the Study group and each of the three Comparison Groups. We estimated a propensity score for the risk of an individual contracting COVID-19 illness based upon previously defined, multivariable predictive equations that take into account age, sex, race/ethnicity, a longitudinal comorbidity score, an outpatient physiology-based severity-of-illness score, and neighborhood deprivation index (24). We used this propensity score to match patients from the Study group to patients within each of the three Comparator groups in a 1:1 fashion, based on a greedy matching algorithm (25) using the caliper method and a maximum absolute difference of 0.005 in the propensity score, with additional hard matching on age (±1 y), hypertension status, diabetes status, and BMI (±3), to further balance the matched groups on factors that have been demonstrated to increase risk for severe COVID-19 illness. Separate matches were done for comparisons of Study Population to each of the three Comparator Groups. We verified the performance of each of the matches by comparing the distribution of covariates between groups both before and after matching.

### Statistical Analysis.

Analyses were performed using SAS software, version 9.4. Crude event rates and incidence rates per 100 person-years for each outcome were calculated using the observed time at risk within each group. For the outcomes reflecting severe COVID-19 illness (hospitalization for COVID-19 and hospitalization for COVID-19 requiring ICU treatment), we calculated incidence rates using two approaches: 1) as a proportion of the total number of members within the study subgroup, and 2) as a proportion of total observed positive COVID-19 cases within the study subgroup. Incidence rate ratios comparing the outcome rate in the Study Population to each of the Comparator Groups were calculated with corresponding 95% confidence intervals (CIs) based on Poisson rates (26).

## Results

**Fig. 1.**
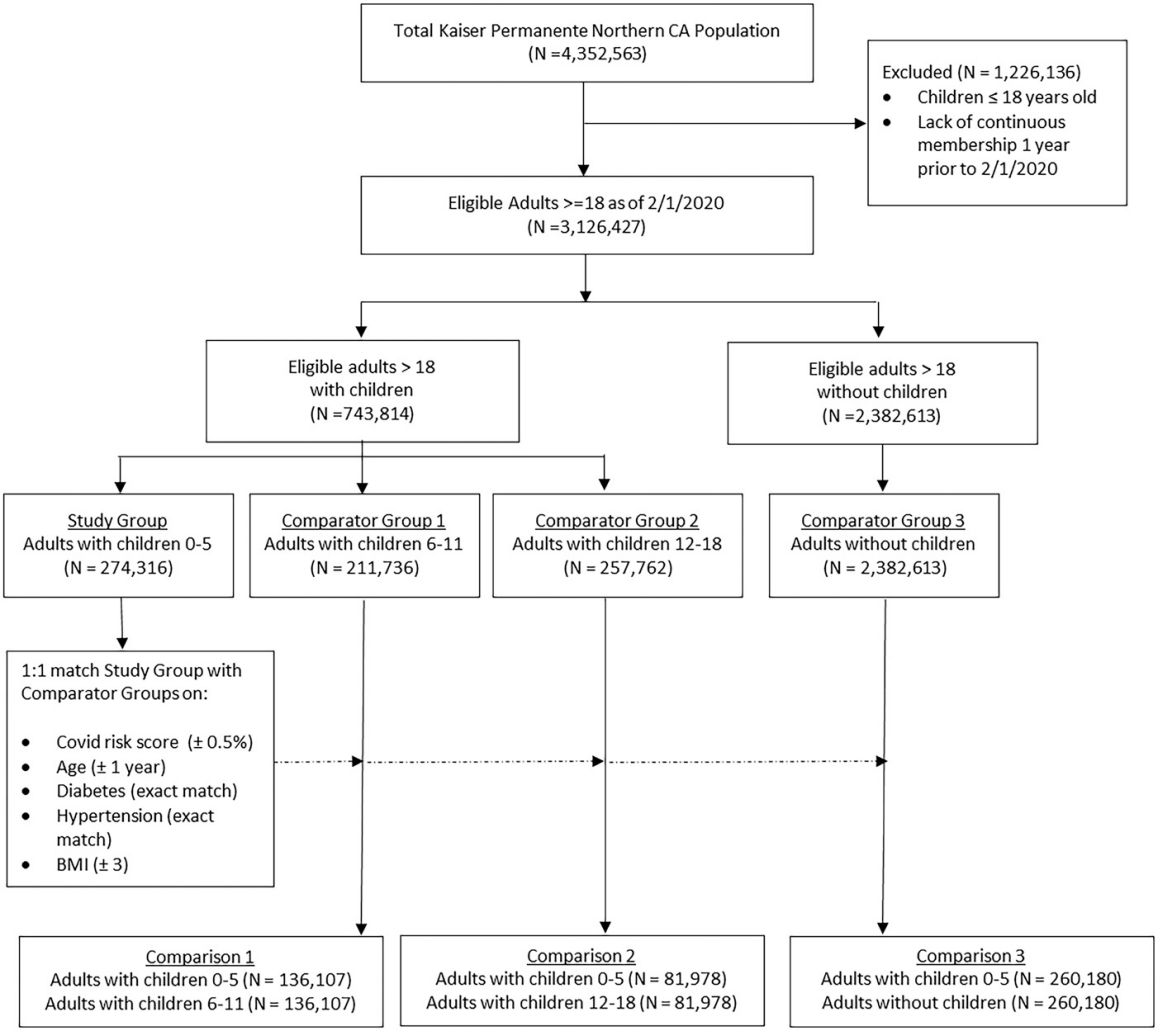
Cohort assembly diagram.

After application of our eligibility criteria, we identified 3,126,427 adults, of whom 24% (*n* = 743,814) had children less than 18 y on their health plan (Fig. 1). After classifying adults into categories based upon the age of the youngest child on the same health plan in the 2 y prior to February 1, 2020, there were 274,316 adults (8.8%) with youngest child 0–5 y, 211,736 adults (6.8%) with youngest child 6–11 y, 257,762 adults (8.2%) with youngest child 11–18 y, and 2,382,613 adults (76.2%) with no children (corresponding to 265,213, 205,744, 250,475, and 2,282,236 person-years of follow-up, respectively).

The average age of adults within each category increased as the age of the associated children increased (mean [SD] 36.2 [6.2] y for adults with children 0–5 y; 41.4 [7.9] y for adults with children 6–11 y; and 44.6 [11.3] y for adults with children 12–18 y) and was highest in the category of adults without children (mean [SD] 50.8 [19.3] y for adults without children) (Table 1). Other demographic parameters and comorbidity profiles were typical for the population and reflective of members in the surrounding Northern California region (19). Of note, rates of risk factors for severe COVID-19 illness, such as hypertension and diabetes, were higher among patients in categories associated with children of older ages. BMI was similar across all categories.

**Table 1.** Baseline characteristics of Study and Comparator cohorts, unadjusted and propensity-matched

	Unadjusted				Propensity matched								
	Study population	Comparator groups			Comparison 1			Comparison 2			Comparison 3		
	Adults with children 0–5 y	Adults with children 6–11 y	Adults with children 12–18 y	Adults without children	Adults with children 0–5 y	Adults with children 6–11 y	*P*	Adults with children 0–5 y	Adults with children 12–18 y	*P*	Adults with children 0–5 y	Adults without children	*P*
	(*n* = 274,316)	(*n* = 211,736)	(*n* = 257,762)	(*N* = 2,382,613)	(*n* = 136,107)	(*n* = 136,107)		(*n* = 81,978)	(*n* = 81,978)		(*n* = 260,180)	(*n* = 260,180)	
Demographic characteristics													
Mean (SD) age, y	36.2 (6.2)	41.4 (7.9)	44.6 (11.3)	50.8 (19.3)	38.9 (6.9)	39.0 (7.0)	0.01	40.0 (8.5)	40.1 (8.6)	0.05	36.2 (6.2)	36.2 (6.2)	1.00
18–29	34,837 (12.7)	16,664 (7.9)	41,201 (16.0)	464,263 (19.5)	12,327 (9.1)	12,327 (9.1)	0.03	12,550 (15.3)	12,551 (15.3)	0.03	32,348 (12.4)	32,347 (12.4)	1.00
30–39	167,578 (61.1)	58,216 (27.5)	16,630 (6.5)	328,738 (13.8)	55,821 (41.0)	55,042 (40.4)		15,710 (19.2)	15,641 (19.1)		159,058 (61.1)	159,059 (61.1)	
40–49	64,991 (23.7)	110,876 (52.4)	101,910 (39.5)	258,344 (10.8)	61,376 (45.1)	62,048 (45.6)		47,112 (57.5)	46,847 (57.2)		62,152 (23.9)	62,152 (23.9)	
50–59	6,098 (2.2)	23,586 (11.1)	87,269 (33.9)	429,599 (18.0)	5,824 (4.3)	5,951 (4.4)		5,829 (7.1)	6,167 (7.5)		5,829 (2.2)	5,829 (2.2)	
60+	812 (0.3)	2,394 (1.1)	10,752 (4.2)	901,669 (37.8)	759 (0.6)	739 (0.5)		777 (1.0)	772 (0.9)		793 (0.3)	793 (0.3)	
Women, *N* (%)	141,080 (51.4)	108,310 (51.2)	132,617 (51.5)	1,250,584 (52.5)	66,934 (49.2)	75,779 (55.7)	<0.001	37,938 (46.3)	47,842 (58.4)	<0.001	138,225 (53.1)	135,047 (51.9)	<0.001
Race, *N* (%)													
White	104,794 (38.2)	78,634 (37.1)	103,171 (40.0)	1,123,542 (47.2)	50,881 (37.4)	50,515 (37.1)	<0.001	29,437 (35.9)	30,141 (36.8)	<0.001	101,873 (39.2)	105,234 (40.5)	<0.001
Black/African American	13,576 (5.0)	12,909 (6.1)	16,831 (6.5)	160,972 (6.8)	7,504 (5.5)	8,576 (6.3)		5,273 (6.4)	6,030 (7.4)		13,180 (5.1)	17,936 (6.9)	
Asian/Pacific Islander	73,074 (26.6)	52,905 (25.0)	58,087 (22.5)	453,222 (19.0)	35,180 (25.9)	33,690 (24.8)		19,133 (23.3)	17,834 (21.8)		71,107 (27.3)	64,463 (24.8)	
Other	10,942 (4.0)	8,510 (4.0)	10,726 (4.2)	111,221 (4.7)	5,520 (4.1)	5,754 (4.2)		3,510 (4.3)	3,635 (4.4)		10,774 (4.1)	8,829 (3.4)	
Unknown	71,930 (26.2)	58,778 (27.8)	68,947 (26.8)	533,656 (22.4)	37,022 (27.2)	37,572 (27.6)		24,625 (30.0)	24,338 (29.7)		63,246 (24.3)	63,718 (24.5)	
Hispanic ethnicity, *N* (%)	61,918 (22.6)	52,082 (24.6)	63,085 (24.5)	456,332 (19.2)	36,509 (26.8)	36,510 (26.8)	1.00	24,476 (29.9)	24,477 (29.9)	1.00	59,897 (23.0)	59,896 (23.0)	1.00
Body Mass Index													
Mean (SD)	28.3 (5.9)	28.7 (6.1)	28.8 (6.2)	28.2 (6.3)	28.8 (6.1)	28.8 (6.1)	0.53	29.2 (6.2)	29.2 (6.3)	0.84	28.3 (5.9)	28.3 (5.9)	0.99
Behaviors													
1-y smoking status, *N* (%)													
Current smoker	8,054 (2.9)	8,109 (3.8)	10,131 (3.9)	116,458 (4.9)	4,282 (3.2)	5,883 (4.3)	<0.001	2,829 (3.5)	4,263 (5.2)	<0.001	7,866 (3.0)	15,465 (5.9)	<0.001
Former smoker	31,381 (11.4)	24,658 (11.7)	29,384 (11.4)	440,556 (18.5)	17,714 (13.0)	16,299 (12.0)		10,986 (13.4)	9,835 (12.0)		31,186 (12.0)	33,526 (12.9)	
Nonsmoker	156,755 (57.1)	113,647 (53.7)	140,077 (54.3)	1,250,838 (52.5)	78,153 (57.4)	75,705 (55.6)		46,205 (56.4)	44,535 (54.3)		154,996 (59.6)	140,648 (54.1)	
Unknown	78,126 (28.5)	65,322 (30.9)	78,170 (30.3)	574,761 (24.1)	35,958 (26.4)	38,220 (28.1)		21,958 (26.8)	23,345 (28.5)		66,132 (25.4)	70,541 (27.1)	
Comorbidities, *N* (%)													
Hypertension	8,324 (3.0)	11,565 (5.5)	21,927 (8.5)	433,895 (18.2)	5,965 (4.4)	5,965 (4.4)	1.00	4,631 (5.7)	4,631 (5.7)	1.00	8,297 (3.2)	8,297 (3.2)	1.00
Diabetes	5,391 (2.0)	7,357 (3.5)	14,410 (5.6)	243,730 (10.2)	3,994 (2.9)	3,994 (2.9)	1.00	3,174 (3.9)	3,174 (3.9)	1.00	5,375 (2.1)	5,375 (2.1)	1.00
Chronic pulmonary disease	12,112 (4.4)	9,388 (4.4)	12,930 (5.0)	188,105 (7.9)	6,424 (4.7)	6,163 (4.5)	0.02	4,062 (5.0)	4,213 (5.1)	0.09	12,069 (4.6)	12,595 (4.8)	<0.001
Peripheral vascular disorder	570 (0.2)	1,164 (0.6)	2,975 (1.2)	212,206 (8.9)	451 (0.3)	484 (0.4)	0.28	385 (0.5)	432 (0.5)	0.10	567 (0.2)	699 (0.3)	<0.001
Chronic kidney disease	605 (0.2)	931 (0.4)	1,966 (0.8)	105,234 (4.4)	446 (0.3)	450 (0.3)	0.89	356 (0.4)	409 (0.5)	0.05	604 (0.2)	839 (0.3)	<0.001
Malignancy	881 (0.3)	1,345 (0.6)	2,479 (1.0)	52,177 (2.2)	565 (0.4)	731 (0.5)	<0.001	394 (0.5)	526 (0.6)	<0.001	880 (0.3)	939 (0.4)	0.17
Cerebrovascular disease	339 (0.1)	466 (0.2)	915 (0.4)	41,179 (1.7)	222 (0.2)	237 (0.2)	0.48	167 (0.2)	215 (0.3)	0.01	339 (0.1)	438 (0.2)	<0.001
Congestive heart disease	161 (0.1)	226 (0.1)	502 (0.2)	32,202 (1.4)	116 (0.1)	109 (0.1)	0.64	94 (0.1)	87 (0.1)	0.6	159 (0.1)	222 (0.1)	0.001
Rheumatologic disease	674 (0.3)	739 (0.4)	1,259 (0.5)	22,845 (1.0)	354 (0.3)	471 (0.4)	<0.001	216 (0.3)	375 (0.5)	<0.001	674 (0.3)	804 (0.3)	<0.001
Myocardial infarction	137 (0.1)	304 (0.1)	658 (0.3)	23,687 (1.0)	108 (0.1)	135 (0.1)	0.08	89 (0.1)	122 (0.2)	0.02	137 (0.1)	160 (0.1)	0.18
Dementia	10 (0.0)	19 (0.0)	40 (0.0)	18,524 (0.8)	4 (0.0)	8 (0.0)	0.25	2 (0.0)	8 (0.0)	0.06	10 (0.0)	28 (0.0)	0.003
Metastatic solid tumor	184 (0.1)	336 (0.2)	636 (0.3)	11,966 (0.5)	126 (0.1)	176 (0.1)	0.004	89 (0.1)	138 (0.2)	0.001	183 (0.1)	186 (0.1)	0.88
Liver disease	128 (0.1)	184 (0.1)	348 (0.1)	8,975 (0.4)	80 (0.1)	93 (0.1)	0.32	66 (0.1)	83 (0.1)	0.16	128 (0.1)	160 (0.1)	0.06
Hemiplegia or paraplegia	65 (0.0)	77 (0.0)	143 (0.1)	4,922 (0.2)	33 (0.0)	48 (0.0)	0.1	32 (0.0)	42 (0.1)	0.24	65 (0.0)	201 (0.1)	<0.001
HIV/AIDS	20 (0.0)	38 (0.0)	53 (0.0)	4,015 (0.2)	13 (0.0)	18 (0.0)	0.37	11 (0.0)	14 (0.0	0.55	20 (0.0)	262 (0.1)	<0.001
Peptic ulcer disease	73 (0.0)	82 (0.0)	159 (0.1)	3,362 (0.1)	44 (0.0)	48 (0.0)	0.68	35 (0.0)	43 (0.1)	0.36	72 (0.0)	103 (0.0)	0.02

Propensity-score matching of patients from our Study Population (adults with children aged 0–5) with adults from each of the Comparator Groups improved balance of underlying characteristics and risk factors for severe COVID-19 illness, including average age, hypertension, diabetes, and BMI (Table 1). Propensity-matching between the Study Population and Comparator Groups 1 and 2 (i.e., adults with children in older age categories) yielded matched samples that were sizable but reflected only a portion of the full Study Population (*n* = 136,107 members or 50% of the Study Population for Comparison 1; *n* = 81,978 members or 30% of the Study Population for Comparison 2). However, nearly the entire cohort (95%) of members in the Study Population was successfully 1:1 matched to members of the Comparator 3 group (adults without exposure to children).

In unadjusted analyses, rates of COVID-19 infection were similar across Study and Comparator groups that had exposure to children of any ages (incidence rate ratio [IRR] 0.98 95% CI [0.96–1.01] and IRR 1.00 [0.97–1.02] for adults with children 6–11 y and 12–18 y vs. those with children 0–5 y, respectively), but were lower in adults without exposure to children (IRR 0.72 [0.71–0.73]) (Table 2). In contrast, rates of COVID-19 infection requiring hospitalization and ICU admission were higher in all Comparator Groups compared to the Study Population, regardless of whether they were calculated as a proportion of the population or as a proportion of total COVID-19 cases (e.g., IRR 2.12 [1.86–2.41] and IRR 2.85 [2.02–4.03] when comparing COVID-19 infection requiring hospitalization and COVID-19 infection requiring ICU for adults with children 12–18 vs. adults with children 0–5 y when estimating outcomes as a population rate within the study subgroup) (Table 2).

**Table 2.** Comparison of COVID-19 infection, COVID-19 infection requiring hospitalization, and COVID-19 infection requiring intensive care unit admission among adults with and without prior exposure to children, unadjusted and propensity-matched

	Unadjusted				Propensity matched								
	Study population	Comparator Groups			Comparison 1			Comparison 2			Comparison 3		
	Adults with children 0–5 y	Adults with children 6–11 y	Adults with children 12–18 y	Adults without children	Adults with children 0–5 y	Adults with children 6–11 y	*P*	Adults with children 0–5 y	Adults with children 12–18 y	*P*	Adults with children 0–5 y	Adults without children	*P*
	(*n* = 274,316)	(*n* = 211,736)	(*n* = 257,762)	(*N* = 2,382,613)	(*n* = 136,107)	(*n* = 136,107)		(*n* = 81,978)	(*n* = 81,978)		(*n* = 260,180)	(*n* = 260,180)	
COVID-19 infection													
Events	15,057	11,444	14,114	94,172	7,467	8,111		4,843	5,285		14,444	12,244	
IR (per 100-PYs)	5.49	5.40	5.48	3.95	5.49	5.96		5.91	6.45		5.55	4.71	
IRR	–	0.98	1.00	0.72	–	1.09	<0.0001	–	1.09	<0.0001	–	0.85	<0.0001
95% CI	–	[0.96–1.01]	[0.97–1.02]	[0.71–0.73]	–	[1.05–1.12]		–	[1.05–1.13]		–	[0.83–0.87]	
Severe COVID outcomes estimated as population rates*													
COVID-19 infection requiring hospitalization													
Events	341	459	679	10,521	225	262		172	180		327	414	
IR (per 100-PYs)	0.12	0.22	0.26	0.44	0.17	0.19		0.21	0.22		0.13	0.16	
IRR	–	1.74	2.12	3.55	–	1.16	0.09	–	1.05	0.70	–	1.27	0.0014
95% CI	–	[1.52–2.01]	[1.86–2.41]	[3.19–3.96]	–	[0.97–1.39]		–	[0.85–1.29]		–	[1.1–1.46]	
COVID-19 infection requiring ICU													
Events	44	69	118	1860	36	32		28	25		43	64	
IR (per 100-PYs)	0.016	0.033	0.046	0.078	0.026	0.024		0.034	0.030		0.017	0.025	
IRR	–	2.03	2.85	4.87	–	0.89	0.63	–	0.89	0.68	–	1.49	0.0437
95% CI	–	[1.39–2.97]	[2.02–4.03]	[3.61–6.56]	–	[0.55–1.43]		–	[0.52–1.53]		–	[1.01–2.19]	
Severe COVID outcomes estimated as a proportion of COVID-19 infection cases^†^													
COVID-19 infection requiring hospitalization													
Events	341	459	679	10,521	225	262		172	180		327	414	
IR (per 100-PYs)	2.26	4.01	4.81	11.17	3.01	3.23		3.55	3.41		2.26	3.38	
IRR	–	1.77	2.12	4.93	–	1.07	0.44	–	0.96	0.69	–	1.49	<0.0001
95% CI	–	[1.54–2.04]	[1.87–2.42]	[4.43–5.49]	–	[0.90–1.28]		–	[0.78–1.18]		–	[1.29–1.73]	
COVID-19 infection requiring ICU													
Events	44	69	118	1860	36	32		28	25		43	64	
IR (per 100-PYs)	0.29	0.60	0.84	1.98	0.48	0.39		0.58	0.47		0.30	0.52	
IRR	–	2.06	2.86	6.76	–	0.82	0.41	–	0.82	0.47	–	1.76	0.0043
95% CI	–	[1.41–3.01]	[2.02–4.04]	[5.01–9.11]	–	[0.51–1.32]		–	[0.48–1.40]		–	[1.19–2.58]	

^*^Denominators for incidence rates (IR) and incidence rate ratios (IRR) are based upon total person years of study subgroup.  PY = person-years.  CI = confidence interval.

^†^Denominators for incidence rates (IR) and incidence rate ratios (IRR) are based upon the number of COVID-19 infection cases in the study subgroup.

However, in propensity-matched analyses which balanced risk factors for severe COVID-19 illness, rates of COVID-19 infection were slightly higher for adults with exposure to older children (IRR 1.09 [1.05–1.12] for adults with children 6–11 y and IRR 1.09 [1.05–1.13] for adults with children 12–18 y) compared to those with younger children (0–5 y), although no difference in rates of COVID-19 illness requiring hospitalization or hospitalization requiring ICU admission were observed, using either method of estimating outcomes (i.e., as a proportion of the population or as a proportion of COVID-19 cases within the subgroup) (Table 2). When comparing the Study Population of adults with exposure to children aged 0–5 versus propensity-matched adults without exposure to children, those without exposure to children had lower rates of COVID-19 infection (IRR 0.85 [0.83–0.87]) but significantly higher rates of COVID-19 hospitalization (IRR 1.27 [1.10–1.46]) and hospitalization requiring ICU admission (IRR 1.49 [1.01–2.19]) when calculated as a proportion of the population for each study subgroup, and even higher rates of severe COVID-19 illness when calculated as a proportion of positive COVID-19 cases (IRR 1.49 [1.29–1.73] for COVID-19 hospitalization and IRR 1.76 [1.19–2.58] for hospitalization requiring ICU admission).

## Discussion

We sought to determine whether adults who are at higher likelihood of having prior exposure to non-SARS-CoV-2 coronaviruses may be at decreased risk for severe COVID-19 illness compared to patients at otherwise similar risk of adverse COVID-19 outcomes. To do this, we examined a propensity-matched cohort of patients with prepandemic exposure to young children, a group with the highest rate of upper respiratory infections and the highest likelihood of passing them on to adults (12, 13, 16–18, 27, 28), and compared their rates of COVID-19 infection and rates of severe COVID-19 illness including hospitalization and need for ICU, to adults who did not have the same presumed level of exposure to small children and, by proxy, non-SARS-CoV-2 coronavirus infections. We found that exposure to young children was not associated with a reduction in COVID-19 infection rates, but was associated with protection against severe COVID-19 illness. Those without identifiable household exposure to children based on health insurance enrollment had a 27% higher rate of COVID-19 hospitalization and a 49% higher rate of COVID-19 hospitalization requiring ICU admission than those with young children, when calculating outcomes for severe illness relative to the total population within each subgroup. When severe COVID-19 outcomes were calculated as a proportion of those who contracted COVID-19 infection within each age and exposure group—in other words, the risk of a severe adverse COVID-19 outcome among adults with confirmed COVID-19—the findings were more dramatic, with a 49% higher rate of COVID-19 hospitalization and a 76% higher rate of COVID-19 hospitalization requiring ICU admission among those without exposure to young children. Our findings, based on data prior to the availability of COVID-19 vaccines, provide potential epidemiologic evidence to suggest the possibility that cross-immunity to non-SARS-CoV-2 coronaviruses may provide a level of protection against severe COVID-19 illness.

While we were unable to utilize serum-level assays of immunity or prior infection in our study, our work adds population-level support to the hypothesis that cross-immunity may play a role in the individual response to COVID-19 infection, as exposure to young children is an excellent proxy for prior endemic coronavirus infection. Our results are in line with smaller studies that utilized banked samples from patients with prior endemic coronavirus exposure, and found lower rates of ICU admission and death among those with assay-identified prior coronavirus infection (29), and in vivo studies that suggest that preexisting, cross-reactive memory T-cells may facilitate rapid viral control (4). Other studies did not have adequate sample size to conduct propensity-matched comparisons at a population level that could improve the balance of underlying risk factors, and did not examine outcomes over the full course of the prevaccine period of the pandemic.

Reports from adults in professions with exposure to small children, such as teachers and other school workers, suggest periods of recurrent viral upper respiratory infections at the start of their careers (27)—for example, this is commonly described by pediatrics residents, daycare workers, grade school teachers, as well as young parents (28). Anecdotally, it is reported that after several rounds, or even years, of exposure, recurrent illnesses abate and do not recur, and data suggest that older workers in high-exposure settings do experience fewer seasonal upper respiratory infections (27). It is thought that development of memory immunity, and cross-immunity to families of viruses, may play a role in this cycle. In our study, we attempted to examine this phenomenon at a population-level, to see if a signal for cross-immunity to coronaviruses may protect against COVID-19, by comparing rates of severe COVID-19 illness across cohorts of adults with differential exposure to children.

We did not observe differences in severe COVID-19 illness between adults with children of different age groups. The duration of immunity and potential cross-immunity to prior exposure to non-SARS-CoV-2 coronaviruses, or innate pan-viral protection from recent viral exposures that has been observed (15), is not well understood. It may be the case that adults with exposure to older age children have lasting immune protection against severe illness, despite lower rates of household viral infections as children get older.

Our results may in fact represent underestimates of the impact of cross-immunity. For example, it is possible that adults who were exposed to children may be misclassified by our criteria and labeled as not having exposure to children if they were, for example, divorced or separated and one partner and on a different health insurance plan.

### Limitations.

Our study has several important limitations. First, we are unable to completely identify and control for all confounding factors that may contribute to severe COVID-19 illness. However, we did control and propensity-match for all known and significant risk factors for severe COVID-19 illness, including age, hypertension, diabetes, BMI, and matching with a novel COVID-19 risk score as a propensity variable (24). We also could not control for differences in potential exposure to contracting COVID-19 infection, although, as noted, we did control and balance for known risk factors for severe COVID-19 illness. In addition, when our data on severe COVID-19 outcomes were analyzed as a proportion of confirmed COVID-19 cases, the association of reduced severe COVID-19 illness among those with exposure to young children was only strengthened. Further, there may be misclassification of adults’ exposure to children, although, as noted above, estimates would only be biased downward in our study if children were misclassified such that our Comparator group of adults without children did have exposure to children. Finally, we do not have serum-level data to analyze which families had documented prior exposure to endemic coronaviruses, and thus our results should be considered hypothesis-generating and not definitive. For further evidence of the effect of acquired cross-immunity hypothesis, additional inquires that compare occupations that have significant exposure to younger children could be considered, such as comparing COVID-19 outcomes among pediatricians to geriatricians, or kindergarten teachers to high-school teachers.

### Conclusion.

In summary, in our study of a large, real-world patient population of adults with and without exposure to young children, we found that exposure to young children was not associated with reductions in rates of COVID-19 infection, but was associated with significantly less severe COVID-19 illness. For those without exposure to young children, we observed up to 49% increased risk of hospitalization for COVID-19 and up to 76% increased risk of hospitalization for COVID-19 requiring ICU admission, when balancing known and observable risk factors for severe COVID-19 illness. These results suggest that endemic coronavirus cross-immunity may play a role in protection against severe COVID-19 illness, and may explain some of the observed heterogeneity in the occurrence of adverse COVID-19 outcomes. Future research should examine whether the effect is robust across other types of exposure (e.g., occupational) to young children, and could include more detailed immunologic analyses to test for evidence of cross-immunity.

## Data Availability

All study data are included in the main text.
